# Trends and Patterns in Hypertension-Related Deaths: A Comprehensive Analysis Using Center for Disease Control and Prevention’s Wide-Ranging Online Data for Epidemiologic Research (CDC WONDER) Data

**DOI:** 10.7759/cureus.70754

**Published:** 2024-10-03

**Authors:** Oyinlola O Fasehun, Joycelyn Adjei-Mensah, Wisdom S Ugorji, Victoria O Titus, Oluwatobi O Asade, Damilola A Adeyemo, Okelue E Okobi

**Affiliations:** 1 Internal Medicine, University of Texas Rio Grande Valley, Weslaco, USA; 2 Internal Medicine, Achimota Hospital, Achimota, GHA; 3 General Practice, National Health Service England, Newcastle Upon Tyne, GBR; 4 General Practice, Fell Tower Medical Centre, Newcastle Upon Tyne, GBR; 5 Internal Medicine, Healthy Choice Family Clinic, Largo, USA; 6 Internal Medicine, College of Medicine, Lagos State University, Lagos, NGA; 7 Research, Texas A&M University, Corpus Christi, USA; 8 Family Medicine, Medficient Health Systems, Laurel, USA; 9 Family Medicine, Lakeside Medical Center, Belle Glade, USA; 10 Family Medicine, Larkin Community Hospital, Palm Springs Campus, Miami, USA

**Keywords:** age-specific mortality, cdc wonder, gender disparities, hypertension, mortality, public health, racial disparities

## Abstract

Hypertension (HTN) is a leading cause of cardiovascular morbidity and mortality worldwide. Despite advances in treatment, including the development and use of vasodilator-β-blocker combination and treatment with antihypertensive agents, HTN-related deaths have shown concerning trends. As such, the objective of this study is to examine the trends, disparities, and demographic variations in HTN-related mortality over a decade and to identify key factors contributing to these patterns, including genetics, dietary habits, structural discrimination in access to healthcare, lifestyle choices, and secondary hypertension, which is due to underlying conditions like kidney disease, hormonal disorders, or certain medications. To attain this objective, this retrospective study has utilized data from the Center for Disease Control and Prevention's Wide-Ranging Online Data for Epidemiologic Research (CDC WONDER) database to assess HTN-related mortality rates from 2010 to 2020. Age-adjusted mortality rates were calculated, and subgroup analyses were conducted by gender, race/ethnicity, and age groups. Temporal trends were analyzed to identify significant changes in mortality rates over time. Moreover, IBM SPSS Statistics, version 29 (IBM Corp., Armonk, NY) was used in the analysis, while 95% confidence intervals (CIs), were calculated to demonstrate the temporary trend of mortality rates overall and by age, sex, ethnicity, and region. Therefore, the mortality data from 2010 to 2020 show significant trends and variations across demographic groups. Overall, HTN-related mortality rate in the United States increased from 5.1 per 100,000 in 2010 to 6.4 in 2020, reflecting a general upward trend. For males, the rate rose from 4.8 to 6.6 per 100,000 during the same period. Racial disparities are notable, with Black or African American individuals having the highest mortality rates, increasing from 9.6 to 11.2 per 100,000. Age-specific data reveal that mortality in the 65-74 age group more than doubled, from 10.3 to 16.2 per 100,000, while in the 75-84 age group, it rose from 32.1 to 35.7. The 85+ age group had the highest rates, increasing from 144.0 to 155.0 per 100,000. States with the highest age-adjusted rates include Mississippi, Georgia, West Virginia, California, and Alabama. The study findings highlight the growing burden of HTN-related mortality in the United States, particularly among males, racial minorities, and older adults. This situation underscores the need for targeted public health interventions, which include creation of hypertension awareness in minority groups and enhancing medication adherence especially among Blacks, and addressing the social determinants of health contributing to higher HTN rates and poorer outcomes, including disadvantaged neighborhoods, structural discrimination and racism, and limited access to healthcare. The study found that African Americans are likely to be diagnosed with HTN earlier in life with higher HTN-related mortality than Whites, and with 50% increased risk of cardiovascular disease mortality. Continuous efforts are required to aptly address such disparities contributing to ongoing HTN treatment and care inequalities.

## Introduction

Hypertension (HTN) or high blood pressure (BP), is a global public health challenge that is often underdiagnosed despite its high prevalence rate and significant health implications [1]. HTN is characterized by a consistent elevation of arterial pressure, resulting in an increased workload to the heart of pumping blood throughout the body. The increased workload significantly increases the risk of severe cardiovascular complications, including heart disease, stroke, and kidney failure [2]. Globally, HTN affects approximately 1.28 billion adults aged 30-79 years. Notably, two-thirds of these individuals live in low- and middle-income countries, with limited access to healthcare and effective management. Alarmingly, about 46% of HTN patients are unaware of their condition, while only 42% receive a diagnosis and appropriate treatment. In those treated, only 21% achieve adequate blood pressure control [1-4].

The United States has witnessed a steady increase in HTN prevalence, driven by factors including an aging population, lifestyle changes, and rising obesity rates. According to the American Heart Association, nearly half of all US adults either have HTN or are at significant risk of developing it [3-5]. This increasing HTN prevalence raises public health concern, as HTN is a major risk factor for cardiovascular diseases, a leading cause of morbidity and mortality, globally. Further, the HTN burden is unevenly distributed across the population, with significant disparities observed based on age, gender, race, and socioeconomic status, with the elderly persons, African Americans, and poor populations being the most affected [4,5]. The high prevalence rate is attributable to aspects like lack of access to healthcare and effective HTN management, given that the most affected populations being the elderly persons, African Americans, and individuals of low socioeconomic status. The comprehension of the disparities in HTN mortality trends will enable development of effective targeted public health interventions for the most affected populations, to reduce the rates of HTN-related mortality. 

HTN entails a complex interplay of genetic, environmental, and physiological factors, and has been categorized as either primary HTN, which is more common and influenced by factors like genetics, dietary habits, and lifestyle choices, or secondary HTN, which is less common and attributable to underlying conditions like kidney disease, hormonal disorders, and certain medications [6,7]. HTN’s pathophysiological mechanisms involves complex processes, including endothelial dysfunction, arterial stiffness, and increased cardiac workload. Such changes progressively damage target organs (heart, brain, and kidneys) [1,6,8,9]. This study utilizes the Center for Disease Control and Prevention's (CDC) Wide-ranging Online Data for Epidemiologic Research (WONDER) database to analyze hypertension-related mortality trends in the United States from 2010 to 2020, as the duration offers a better comprehension of HTN mortality trends over the last decade to enable better projection of the trends and development of effective public health interventions. For this study, CDC WONDER database has been chosen as it provides a menu-driven access to around 24 databases with data on mortality, morbidity, prevalence rates on a regular basis, in addition to promoting information-driven decision making. Therefore, the primary objective is to identify significant trends in hypertension-related mortality and explore disparities by gender and race, and assess geographic variations [10].

## Materials and methods

Study design and data source, Inclusion and exclusion criteria

This study is a retrospective analysis of HTN-related mortality trends in the United States from 2010 to 2020. The data were obtained from the CDC WONDER database. This comprehensive, publicly accessible resource aggregates extensive health-related information, including mortality statistics derived from death certificates provided by the National Vital Statistics System (NVSS). The data drawn from the CDC WONDER database was evaluated with the objective of assessing the epidemiological trends and patterns associated with mortality rates in persons with HTN and HTN-related conditions. The study population comprised persons who had been diagnosed with HTN and HTN-related conditions and who died because of these conditions. The database mainly focused on mortality data acquired from death certificates, offering important information regarding the place of death and various patient-specific demographics that include details regarding the death cause, demographic factors, and other pertinent information. Further, the study included all deaths in the United States during the specified period where HTN was recorded as either an underlying or contributing cause of death. Cases were selected based on the identified ICD-10 codes. Exclusion criteria were applied to deaths with incomplete demographic data (missing data on a variable, particularly with regard to the demographics of the patient) or those with unreliable reported data (data drawn from the patients/participants that is inaccurate and untrustworthy). This ensured that the analysis was focused on reliable and relevant data, enhancing the accuracy of the study's findings.

Data source

The CDC WONDER database, a publicly accessible virtual resource, was the principal source of data for this study. The database contains a wider array of mortality data that include information pertaining to the cause of death, sex, age, geographic location, and notable demographic variables that cover a representative and increasingly diverse sample of the US population. The study assessed all-cause mortality along with the cause-specific mortality rates for HTN-related conditions, using the more specific International Classification of Disease (ICD-10) codes: code I10 (primary HTN), I11 (hypertensive heart disease), I12 (hypertensive chronic kidney disease), I13 (hypertensive heart and chronic kidney disease), and I15 (Secondary hypertension) [10,11]. Mortality cases that had incomplete or unreliable data were excluded, with the mean/median imputation methodology being used in addressing the missing data.

Study variables, data extraction, and data analysis

HTN-related mortality data were extracted for each year within the study period, focusing on variables such as the number of deaths, age-adjusted death rates per 100,000 population, and key demographic characteristics, including age, sex, race/ethnicity, and geographic region at the state level. This comprehensive approach allowed for a detailed exploration of trends and disparities in HTN-related mortality across different population subgroups and geographic areas. The analysis was conducted in several stages. First, descriptive statistics were used to calculate the total number of HTN-related deaths per year and to summarize the age-adjusted death rates. Demographic characteristics of the deceased, including age, sex, race/ethnicity, and geographic location, were also summarized to provide context for the mortality trends observed. Temporal trends in HTN-related mortality were analyzed using linear graphical representations and descriptive statistics to identify significant changes over time. Trend analysis was utilized in analyzing the distribution of HTN-related deaths alongside the changes in HTN mortality rates trends. Additionally, linear regression was also utilized as the statistical method for analyzing the mortality trends. The study further stratified these trends by age group (e.g., 18-44, 45-64, 65+), sex, race/ethnicity (e.g., non-Hispanic White, non-Hispanic Black, Hispanic, Asian/Pacific Islander), and geographic region (e.g., Northeast, Midwest, South, West) to uncover disparities among different population subgroups. Geographic variations were also examined at the state level, with states ranked by their cumulative age-adjusted mortality rates, based on a 10-year rate, to pinpoint regions with notably high or low HTN-related mortality. Thus, adjusting for age when comparing the state-to-state differences in HTN-related mortality rates is vital, given that age is a dominant risk factor for HTN mortality, even as state age distributions significantly vary. Data analysis was performed using IBM SPSS Statistics, v.29 (IBM Corp., Armonk, NY, USA), ensuring robust and reliable results.

Ethical considerations

The study utilized publicly available, de-identified data, so it was exempt from institutional review board (IRB) approval. The analysis was conducted in accordance with all relevant guidelines and regulations governing the use of health-related information, ensuring ethical handling of the data.

## Results

The mortality data from 2010 to 2020 reveal significant trends and variations across different demographic groups. This analysis highlights key observations regarding overall mortality rates and differences by gender, race, and age. From 2010 to 2020, the overall mortality rate in the United States increased. In 2010, the mortality rate was 5.1 per 100,000 people (95% CI: 5.1-5.2), and it remained stable at 5.1 (5.1-5.2) in 2011. However, from 2012 onwards, there was a notable upward trend. By 2013, the rate had increased to 5.5 (5.4-5.6) and reached 6.4 (6.3-6.5) by 2020. The rise in mortality rates reflects a steady increase in the number of deaths per capita over the decade, with a marked escalation in the last few years. This has been aptly captured in Table 1, which indicates the patterns of underlying causes of death due to HTN, between 2010 and 2020.

**Table 1:** Patterns of underlying causes of death due to HTN (2010-2020) Unit of measurement: For CDC WONDER project, unit of measurement is mortality rate per 100,000 HTN: Hypertension; CI: confidence interval; M: million

Variables	Mortality Rate per 100,000											
	Years	2010	2011	2012	2013	2014	2015	2016	2017	2018	2019	2020
	Total deaths	17265	17818	19215	19964	19518	20696	21276	22598	22905	23056	26789
	Percentage increase in mortality rates	17265 (0%)	553 (3.20%)	1,397 (7.84%)	749 (3.75%)	-446 (-2.23%)	1178 (6.04%)	580 (2.80%)	1322 (6.21%)	307 (1.36%)	151 (0.66%)	3733 (16.19%)
National Data	Total population	308M	311M	313M	316M	318M	321M	323M	325M	327M	328M	329M
	Overall mortality rate	5.1	5.1	5.4	5.5	5.3	5.4	5.5	5.7	5.7	5.6	6.4
	95% CI	5.1-5.2	5.1-5.2	5.3-5.5	5.4-5.6	5.2-5.3	5.4-5.5	5.4-5.6	5.6-5.8	5.6-5.8	5.5-5.7	6.3-6.5
Based on gender	Male	4.9	4.8	5.1	5.3	5.2	5.4	5.5	5.9	5.8	5.8	6.6
	95% CI	4.7-5	4.7-4.9	5-5.2	5.2-5.4	5.1-5.3	5.3-5.5	5.4-5.6	5.7-6	5.7-5.9	5.7-5.9	6.5-6.7
	Female	5.2	5.1	5.4	5.4	5.1	5.3	5.3	5.4	5.4	5.3	6
	95% CI	5.1-5.3	5-5.2	5.3-5.5	5.3-5.5	5-5.2	5.2-5.4	5.2-5.4	5.3-5.5	5.3-5.5	5.2-5.4	5.9-6.1
Based on race	White	4.7	4.8	5	5.1	4.9	5.1	5.1	5.3	5.3	5.2	5.9
	95% CI	4.6-4.8	4.7-4.9	4.9-5.1	5.1-5.2	4.9-5	5-5.2	5.1-5.2	5.3-5.4	5.2-5.4	5.1-5.3	5.8-6
	Black/African American	9.6	8.9	9.3	9.3	8.8	9.2	9	9.3	9.2	9.4	11.2
	95% CI	9.2-10	8.6-9.3	9-9.7	8.9-9.6	8.5-9.1	8.8-9.5	8.7-9.3	8.9-9.6	8.9-9.5	9.1-9.7	10.9-11.6
	Asian/Pacific Islander	4.4	4.2	4.2	4.2	3.9	4.3	4.5	4.9	4.9	4.6	5.3
	95% CI	4-4.8	3.8-4.6	3.9-4.6	3.8-4.5	3.6-4.2	3.9-4.6	4.2-4.8	4.6-5.2	4.6-5.2	4.3-4.9	5-5.6
	American Indian/Alaska Native	3.5	3.2	3.9	4.5	3.8	3.6	3.5	3.7	3.6	3.3	4.6
	95% CI	2.7-4.5	2.5-4.2	3.1-4.8	3.6-5.3	3-4.5	2.9-4.5	2.8-4.2	3-4.4	2.9-4.2	2.6-3.9	3.9-5.4
Based on age	25-34 years	0.1	0.1	0.2	0.1	0.2	0.2	0.2	0.2	0.2	0.1	0.2
	95% CI	0.1-0.1	0.1-0.1	0.1-0.2	0.0-0.0	0.1-0.2	0.1-0.2	0.1-0.2	0.1-0.2	0.1-0.2	0.0-0.1	0.1-0.2
	35-44 years	0.5	0.6	0.5	0.6	0.7	0.7	0.8	0.8	0.7	0.7	0.9
	95% CI	0.5-0.6	0.5-0.7	0.5-0.6	0.5-0.7	0.6-0.7	0.7-0.8	0.7-0.8	0.7-0.9	0.6-0.8	0.6-0.8	0.8-1.0
	45-54 years	2.1	2.1	2.1	2.5	2.4	2.4	2.6	2.6	2.7	2.6	3.1
	95% CI	1.9-2.2	1.9-2.2	1.9-2.2	2.3-2.6	2.2-2.5	2.3-2.6	2.4-2.7	2.5-2.8	2.5-2.8	2.4-2.7	2.9-3.2
	55-64 years	4.7	4.8	5.5	5.8	5.9	6.3	6.6	6.8	7.0	6.9	8.5
	95% CI	4.5-5.0	4.6-5.0	5.3-5.8	5.5-6.0	5.7-6.2	6.1-6.6	6.3-6.8	6.6-7.1	6.8-7.3	6.7-7.2	8.2-8.8
	65-74 years	10.3	10.8	10.8	11.4	11.0	11.6	12.5	12.9	13.2	13.6	16.2
	95% CI	9.8-10.7	10.4-11.2	10.4-11.3	11.0-11.8	10.6-11.4	11.2-12.0	12.1-12.9	12.5-13.3	12.8-13.6	13.2-14.0	15.8-16.6
	75-84 years	32.1	30.6	32.2	32.7	31.5	32.7	31.8	33.6	33.1	32.2	35.7
	95% CI	31.1-33.0	29.7-31.6	31.3-33.2	31.8-33.7	30.6-32.5	31.8-33.7	30.8-32.7	32.6-34.5	32.2-34	31.4-33.1	34.8-36.7
	85+ years	144	144.8	152.9	149.5	138.9	141.7	140.5	146.1	143	140.5	155
		140.8-147.2	141.7-148	149.7-156	146.4-152.6	135.9-141.8	138.7-144.6	137.6-143.4	143.1-149	140.1-145.9	137.6-143.4	152-158

HTN-related mortality rate based on gender

Over this 11-year period, the total number of HTN-related deaths across both genders increased. The mortality rate of males was 4.8 per 100,000 (95% CI: 4.7-5.0) in 2010, which remained stable at 4.8 (4.7-4.9) in 2011. However, this rate gradually increased each subsequent year, reaching 6.6 (6.5-6.7) by 2020. This upward trend signifies a notable rise in mortality rates among males over the decade, particularly in the latter years. In contrast, females had a mortality rate of 5.2 per 100,000 (95% CI: 5.1-5.3) in 2010, which remained relatively stable, with minor fluctuations, culminating in a rate of 6.0 (5.9-6.1) by 2020. While this reflects a smaller increase compared to males, there is still a noticeable upward trend over the period.

**Figure 1:**
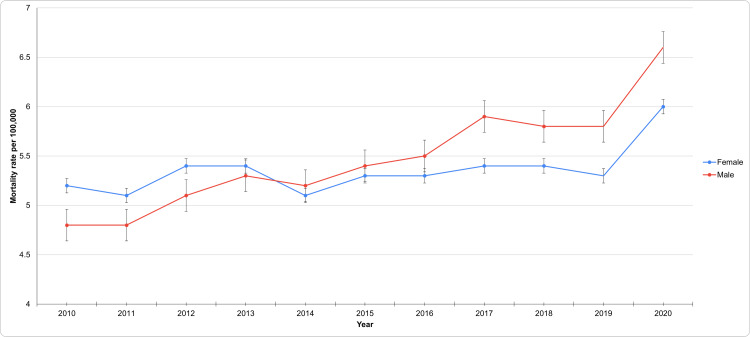
Patterns of underlying causes of death due to HTN based on gender HTN: Hypertension

Throughout the decade, the HTN-related mortality in both genders was rising, but the patterns of increase differed. For females, the age-adjusted rate fluctuated between 5.1 and 5.4 per 100,000 from 2010 to 2017 before rising more sharply in the later years. In contrast, the age-adjusted rate for males demonstrated a more consistent increase, particularly from 2015 onwards, culminating in a significant rise to 6.6 per 100,000 by 2020. These findings suggest that while HTN-related mortality increased in both genders, but it was a steeper rise in males, leading to a narrowing gender gap in mortality rates over time. This has been aptly captured in Figure 1, which indicates the patterns of underlying causes of death due to HTN based on gender.

HTN-related mortality rate based on race

**Figure 2:**
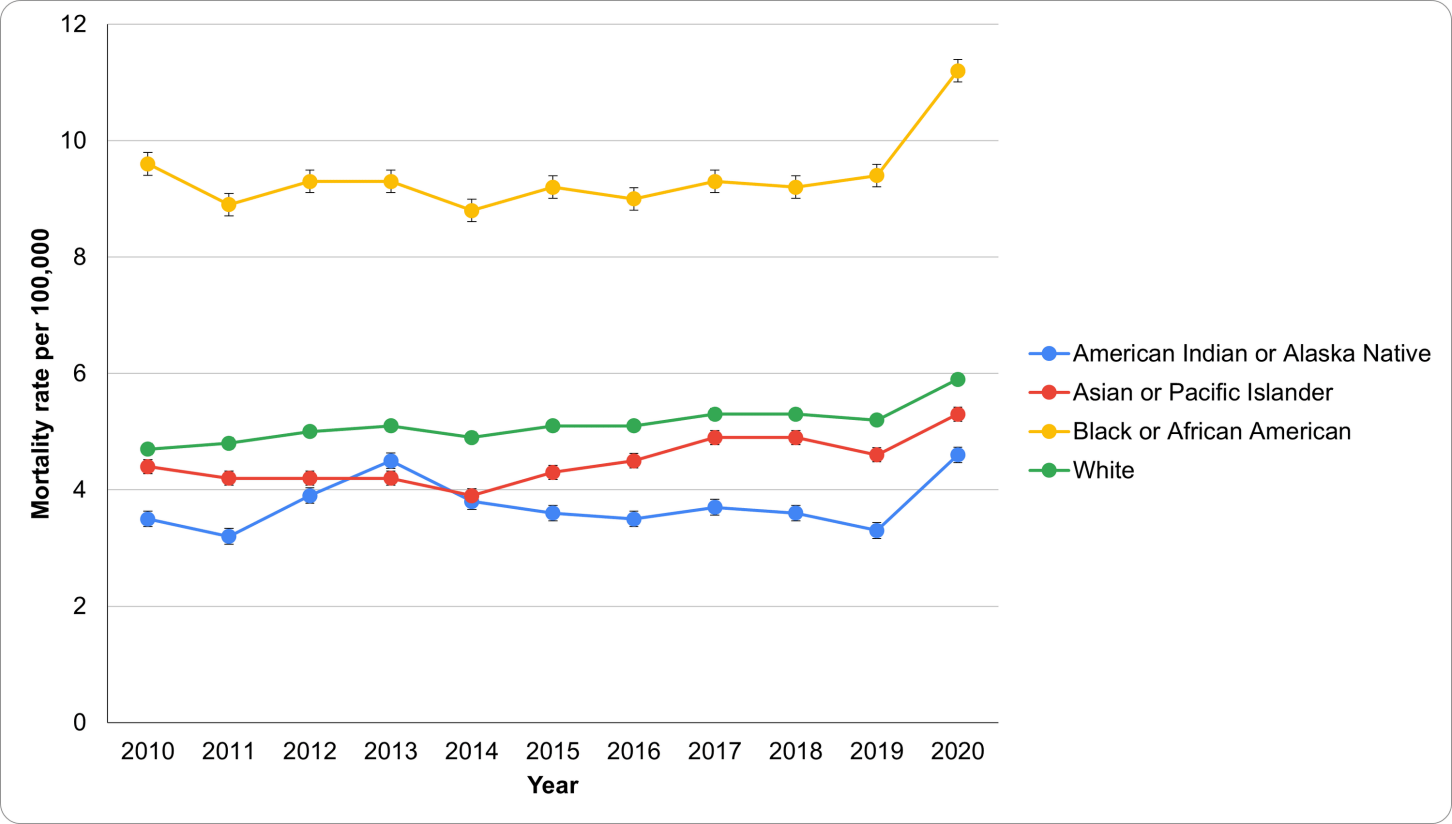
Patterns of underlying causes of death due to HTN based on race HTN: Hypertension

From 2010 to 2020, significant disparities in HTN-related mortality rates emerged among different racial groups (Figure 2). The Black or African American population consistently had the highest age-adjusted mortality rates, starting at 9.6 per 100,000 (95% CI: 9.2-10.0) in 2010 and rising to 11.2 (95% CI: 10.9-11.6) in 2020. This increase highlights a pressing public health concern, necessitating targeted interventions to address the social determinants affecting this community. In contrast, the White population saw a gradual rise in HTN-related mortality, from 4.7 (95% CI: 4.6-4.8) per 100,000 in 2010 to 5.9 (95% CI: 5.8-6.0) in 2020. While lower than that of Black or African Americans, this trend indicates an increasing significance of HTN as a cause of death.

The Asian or Pacific Islander group had relatively stable HTN-related mortality rates, slightly increasing from 4.4 (95% CI: 4.0-4.8) per 100,000 in 2010 to 5.3 (95% CI: 5.0-5.6) in 2020, suggesting a need for focused public health initiatives. Lastly, the American Indian or Alaska Native population had the lowest HTN-related mortality rates, beginning at 3.5 (95% CI: 2.7-4.5) per 100,000 in 2010 and rising to 4.6 (95% CI: 3.9-5.4) by 2020. Despite being the lowest, this increase underscores the importance of continuous monitoring and targeted efforts to prevent further escalation in HTN-related mortality among this group. The patterns of underlying causes of death due to HTN based on race have been aptly captured in Figure 2.

Mortality by HTN across age groups

**Figure 3:**
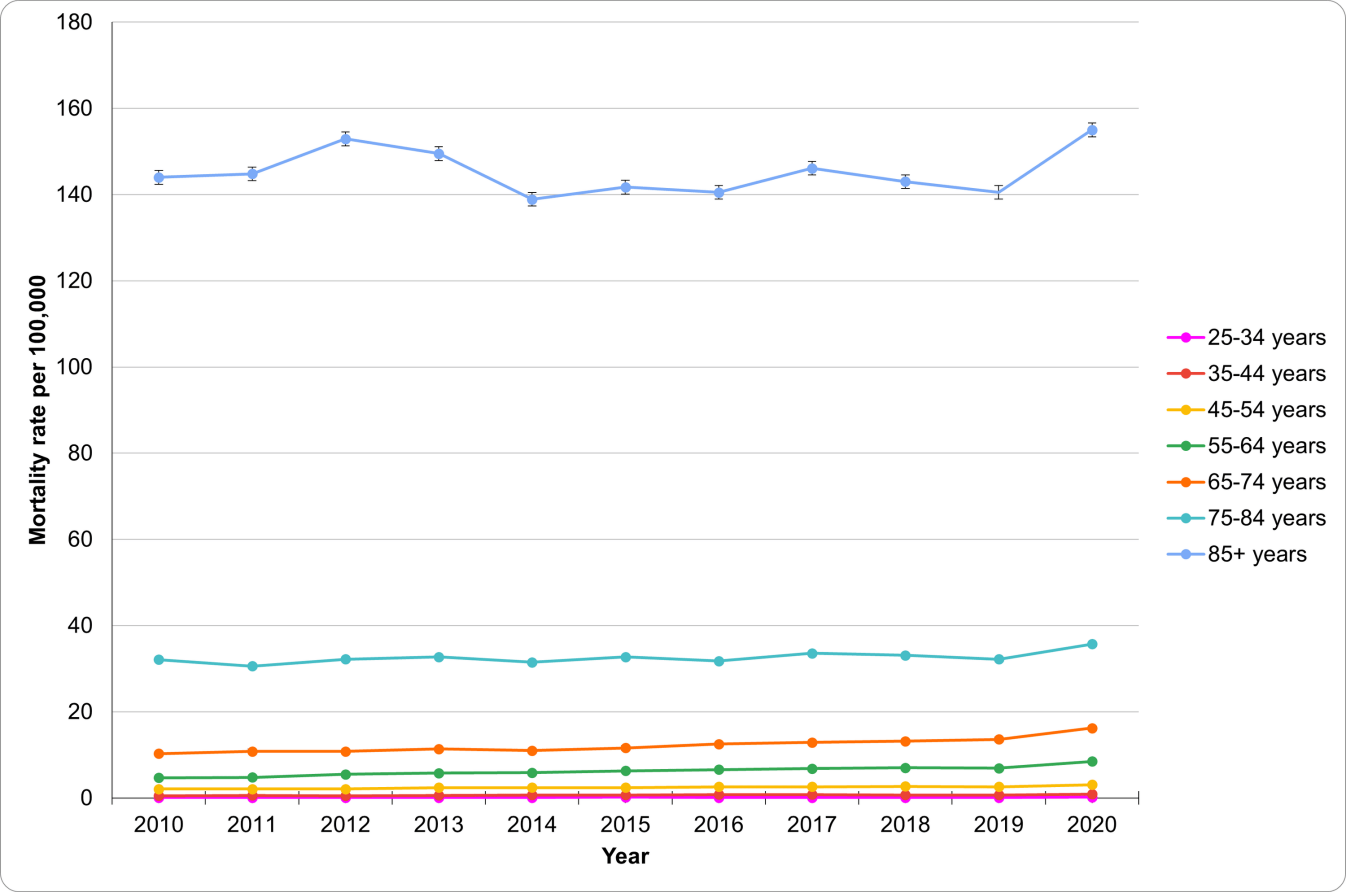
Patterns of underlying causes of death due to HTN based on age groups HTN: Hypertension

From 2010 to 2020, HTN-related mortality increased across all age groups, with the most significant rise in older adults (Figure 3). The 25-34 age group maintained relatively low rates, ranging from 0.1 (95% CI: 0.1-0.1) per 100,000 in 2010 to 0.2 (95% CI: 0.1-0.2) in 2020. The most notable increase occurred between 2019 and 2020, from 0.1 (95% CI: 0.0-0.1) to 0.2, rising by 19%. In the 35-44 age group, mortality exhibited an upward trend, increasing from 0.5 (95% CI: 0.5-0.6) per 100,000 in 2010 to 0.9 (95% CI: 0.8-1.0) in 2020, reflecting a 34% rise from 2019 to 2020. The mortality rate in the 45-54 age group also gradually increased, rising from 2.1 (95% CI: 1.9-2.2) to 3.1 (95% CI: 2.9-3.2) over the decade, representing a 34% increase. The rise was more pronounced in the 55-64 age group, with mortality increasing from 4.7 (95% CI: 4.5-5.0) in 2010 to 8.5 (95% CI: 8.2-8.8) in 2020. The most significant jump occurred between 2019 and 2020, rising from 7.0 (95% CI: 6.8-7.3) to 8.5, a 23% increase. Mortality in the 65-74 age group registered a 57% increase, rising from 10.3 (95% CI: 9.8-10.7) to 16.2 (95% CI: 15.8-16.6). The 75-84 age group showed a consistent rise from 32.1 (95% CI: 31.1-33.0) to 35.7 (95% CI: 34.8-36.7), while the 85+ age group exhibited the highest rates, increasing from 144.0 (95% CI: 140.8-147.2) to 155.0 (95% CI: 152.0-158.0). These findings highlight the growing impact of HTN on older adults across all age groups. The patterns of underlying causes of death due to HTN based on age groups have been aptly captured in Figure 3.

Hypertension mortality by states

**Figure 4:**
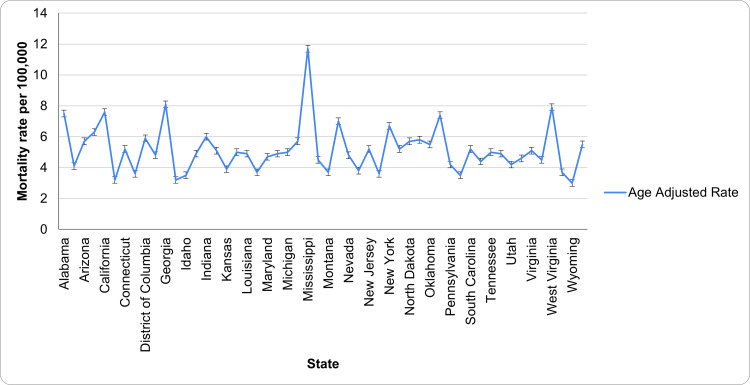
Patterns of underlying causes of death due to HTN based on state HTN: Hypertension

The five states with the highest age-adjusted rates are Mississippi (11.7, CI: 11.3-12.0), Georgia (8.1, CI: 7.9-8.2), West Virginia (7.9, CI: 7.6-8.2), California (7.6, CI: 7.5-7.7), and Alabama (7.5, CI: 7.3-7.7). Mississippi leads with the highest mortality rate due to HTN, significantly above the others. The states with the lowest rates are Wyoming (3.0, CI: 2.6-3.4), Hawaii (3.2, CI: 2.9-3.4), Colorado (3.2, CI: 3.1-3.4), Rhode Island (3.5, CI: 3.2-3.8), and Idaho (3.5, CI: 3.3-3.8). Wyoming registered the lowest rate, indicating a significantly lower occurrence compared to other states. The patterns of underlying causes of death due to HTN based on state have been aptly captured in Figure 4.

## Discussion

The analysis of HTN-related mortality from 2010 to 2020 reveals significant trends, including the increase in HTN mortality rates in elderly persons and individuals aged between 35 and 44 years, among whom a 34% increase in mortality rates was observed between 2019 and 2020, and disparities, with African Americans registering the highest increase in mortality rates, from 9.6 to 11.2 per 100,000 between 2010 and 2020. These findings place emphasis on the ongoing need for public health initiatives to combat this condition. Over the decade, there was a gradual increase in overall mortality rates, reflecting a growing number of deaths linked to HTN amidst population growth. This upward trend signals a notable shift in HTN-related health outcomes, particularly in 2020, possibly influenced by factors such as an aging population, the rising prevalence of chronic diseases, and the exacerbating effects of the COVID-19 pandemic [12,13]. A systematic review by Bajgain et al. identified hypertension, followed by diabetes and cardiovascular diseases, as the most common comorbidities among COVID-19-positive patients globally. The pandemic has been associated with increased mortality rates due to various health complications, including HTN, further underscoring the need for comprehensive management and prevention strategies [14]. Its noteworthy that the effects of COVID-19 pandemic in relation to HTN outcomes for patients have been well documented, with several studies indicating that the HTN-related mortality rates were exacerbated by COVID-19 [12-14]. For instance, it has been shown that after the declaration of COVID-19 as a pandemic, nearly 41% of adult hypertensive patients in the United States avoided/delayed care [12-14], with care avoidance being reported as prevalent in certain primary populations, including communities of color, low-resourced communities, and individuals with comorbid conditions including HTN [14]. Moreover, the findings of recent studies that evaluated changes in HTN control during the COVID-19 pandemic have indicated an incremental increase in BP (by between 1.1 and 2.5 mmHg in systolic and between 0.1 and 0.5 mm Hg in diastolic BP, in the course of the pandemic in comparison to the pre-pandemic period) [12-14].

The study’s findings indicate a substantial increase in HTN-related mortality among males, highlighting a growing burden of HTN in this population [15]. This trend aligns with findings from a U.S. study by Everett and Zajacova, who observed a faster rise in HTN-related mortality among men compared to women. The study also uncovered a significant lack of hypertension awareness, with only 32% of hypertensive women and 25% of hypertensive men aware of their condition. The more pronounced increase in mortality among males may result from factors such as differences in HTN management, health-seeking behaviors, and cardiovascular risk profiles [16]. Further, the observed disparities in HTN-related mortality rates between males and females can be clarified on the basis of access to healthcare. Thus, women are highly likely to visit healthcare providers for regular gynecological and birth control services, which increases the possibility of their blood pressure taken during screening [17-19]. Additionally gender differences with regard to healthcare usage might additionally be due to the consequences of heteronormative masculinity scripts, which dictate males should act tough and not look for help in instances of need. Such gender norms have significant outcomes for men's health and have been found to negatively affect the use of healthcare among men, including the use of preventive healthcare services. 

Racial disparities in HTN-related mortality persist and appear to be worsening. Black or African American individuals consistently exhibited the highest mortality rates, reflecting findings from previous studies, such as Ogunniyi et al.'s 2021 research, which also reported higher HTN-related mortality among Black individuals compared to other racial groups [20,21]. Additionally, Abrahamowicz et al. reported that blood pressure control rates in the United States have declined over the past decade, with racial and ethnic minority groups, particularly non-Hispanic Black individuals, showing significantly lower control rates compared to their non-Hispanic White counterparts [22]. The downstream implications of such racial disparities are substantial given that Blacks have a higher HTN-related mortality rate than Whites, making it important to comprehend the disparities in HTN control and mortality rates and develop effective strategies to tackle them. The disclosure that Blacks have lower HTN control rates is unlikely linked to awareness and treatment rates. The findings are probably secondary to aspects that include health-related, patient-related, and physician-related factors [20-22]. Such disparities are further attributable to socioeconomic inequalities, given that low-income patients are prone to face significant barriers to access healthcare and might not be able to afford the HTN medications. Further, owing to the structural inequalities and systematic discrimination history, Black populations have lower trust in extant healthcare systems, necessitating different approaches to care, including care models shifting HTN management from African Americans into the communities. A notable example is the Los Angeles Barbershop BP [blood pressure] Study, in which African American males who received pharmacist-led HTN treatment within the Black-owned barbershops realized a 20.8 mmHg greater blood pressure reductions at 12 months in comparison to the control arm [22-24]. Therefore, scaling up such a HTN care model nationally presents the potential to prevent one out of every three major adverse cardiovascular events in enrolled African American men, in addition to being cost-effective. Also, the persistent disparity highlights the urgent need for targeted public health interventions to address the social determinants of health contributing to higher HTN rates and poorer outcomes in these populations.

Age-specific mortality data also show a troubling increase across all age groups, with the most significant rises seen in those aged 65 and older. Mortality rates increased by 57% in the 65-74 age group, while the 75-84 age group experienced a consistent rise. The highest mortality rates were observed in individuals aged 85 and older, with a marked increase over the decade. These trends emphasize the escalating impact of HTN-related mortality among older adults, underscoring the need for targeted interventions for this vulnerable population. These findings are consistent with earlier research, such as Ostchega et al.'s 2020 study, which highlighted the disproportionately high burden of HTN-related mortality among older adults [20]. The increasing mortality in older age groups likely stems from the higher prevalence of HTN and comorbid conditions in this population [24]. The differences in age-specific mortality rate observed in this study may be clarified through the observation that elderly persons are at high risk of cardiovascular diseases and event than younger adults, in addition to being increasingly vulnerable the negative effects of blood pressure (BP) lowering [25]. Moreover, arterial stiffness remains a key cause of higher systolic BP (SBP) and pulse pressure (PP), alongside lower diastolic blood pressure (DPB) in older persons. Thus, in older persons, when stiffening occurs in the larger arteries, the aortic reservoir is unable to expand and subsequently store more blood, leading to the reaching of higher pressure during systole, even as the pressure becomes lower during diastole as a result of the lack of adequate blood flow from the reservoir in the course of diastole [25]. Furthermore, such age-related BP changes are significant determinants of all-cause mortality and major cardiovascular disease (CVD) events [23-25]. Therefore, this pronounced rise in mortality among older adults underscores the necessity of tailored interventions that address the unique challenges faced by this age group, including improved HTN management and prevention of complications.

This study's strengths include a comprehensive analysis of HTN-related mortality using a decade-long CDC WONDER dataset, allowing for robust trend and disparity examination. The study's inclusion of age, gender, racial, and geographic dimensions provide a multifaceted understanding of mortality patterns. Consequently, a number of limitations exist, including data accuracy variability across regions and time, potential confounders like healthcare access and socioeconomic factors, and the inability to infer causal relationships due to the cross-sectional nature of the data. Moreover, the study is limited by the focus on HTN-related mortality trends for the United States only, which makes the findings ungeneralizable to HTN-related mortality rates in other countries. Nevertheless, the limitations do not proffer any significant effects on the findings of the study, given that the study's scope focuses on the trends and patterns of HTN-related mortality in the United States. As such, the study findings have offered valuable insights into HTN-related mortality trends and disparities in the United States. Still, confounders such as healthcare access and socioeconomic factors might also influence the results, given that they might result in the under- or over-estimation of the mortality rates due to HTN, which might affect the results of the study. In this regard, future studies focus on the global trends and patterns of HTN-related mortality across various regions and racial groups, as this will not only enable an effective comparison of such rates to the U.S. rates, but also enable the comparison of different HTN control and treatment policies and intervention that might be impactful in reducing the deaths. Additionally, such studies will enable generalization of the findings to different populations across the globe. 

## Conclusions

The decade-long analysis of HTN-related mortality reveals concerning trends and patterns in mortality rates, underscoring the challenges posed by an aging population and the increasing prevalence of chronic diseases. The analysis has disclosed significant effects of racial disparities, gender differences, and age differences in HTN outcomes, particularly with regard to HTN-related mortality rates. Addressing these disparities requires targeted, comprehensive, and localized public health interventions to reduce HTN-related deaths. Interventions like enhanced public awareness and education are crucial, particularly in regions with high mortality rates and among high-risk populations, including Black communities, older persons, and males. By increasing awareness about HTN and its risks, earlier diagnosis and better management can be encouraged. Improved access to healthcare, especially in underserved areas, is also vital to ensuring timely diagnosis, treatment, and management of HTN. Further, the findings of this study have indicated that improvement of HTN control is an essential clinical component to tackling the troubling trends in HTN mortality, particularly in elderly populations, working‐age adults, and minority groups. Owing to the extensive nature of the increases in HTN mortality, national calls toward improvement of hypertension control will inform actions in communities and populations with highest hypertension burden in the United States, including the African American population. Therefore, evidence-based interventions should be executed, adapted, and expanded in divergent contexts across the United States, in addition to making improved hypertension control a national priority and developing an environment capable of supporting hypertension detection, prevention, and control, and improving patient care. Additionally, clinical care optimization is essential for the reduction of hypertension and its control in affected population demographics and populations, and this, in turn, lowers hypertension mortality rates. Three notable approaches that should be considered in enhancing care in communities and populations with increasing hypertension mortality rates include fixed-dose combination medications, improvement of medication adherence, and expansion of telehealth services. Thus, increasing medication adherence is crucial, given that several studies have indicated that only half of HTN patients follow medical advices, with lowest adherence rates reported in African American adults. Still, telehealth should be utilized as it proffers opportunities for improving HTN care, particularly in places with restricted access to healthcare, and this potential has been highlighted by the increased expansion and use of telehealth during the COVID-19 pandemic, owing the potential for long-term support that it offers. Telehealth enables self-measured monitoring of blood pressure, which is an evidence-based strategy that aims to assist patients in managing HTN effectively. Therefore, effective control and prevention of HTN mortality requires the equitable execution of these interventions, given that the disparities in HTN care are mostly driven by the unequal access to healthcare alongside healthy milieus, related to the various social determinants of health that include racial discrimination, stress, and poverty. Concentrating on demographic/population specific social determinants will ascertain equitable improvement in HTN control, prevention, and management for the affected persons and populations. In this regard, future studies should focus on the investigation of medication adherence in populations and demographics with higher rates of HTN mortality given the observation that almost half of individuals with HTN have been reported to be failing in their adherence to the prescribed medication regimen. Consequently, it is vital that the future study focuses on specific populations with higher HTN mortality rates, including the African American populations, as this will enable determination of factors that affect their adherence to HTN medications.
